# NeuroElectro: a window to the world's neuron electrophysiology data

**DOI:** 10.3389/fninf.2014.00040

**Published:** 2014-04-29

**Authors:** Shreejoy J. Tripathy, Judith Savitskaya, Shawn D. Burton, Nathaniel N. Urban, Richard C. Gerkin

**Affiliations:** ^1^Department of Biological Sciences, Carnegie Mellon UniversityPittsburgh, PA, USA; ^2^Center for the Neural Basis of Cognition, Carnegie Mellon UniversityPittsburgh, PA, USA

**Keywords:** neuroinformatics, electrophysiology, database, text-mining, metadata, API, machine learning, natural language processing

## Abstract

The behavior of neural circuits is determined largely by the electrophysiological properties of the neurons they contain. Understanding the relationships of these properties requires the ability to first identify and catalog each property. However, information about such properties is largely locked away in decades of closed-access journal articles with heterogeneous conventions for reporting results, making it difficult to utilize the underlying data. We solve this problem through the NeuroElectro project: a Python library, RESTful API, and web application (at http://neuroelectro.org) for the extraction, visualization, and summarization of published data on neurons' electrophysiological properties. Information is organized both by neuron type (using neuron definitions provided by NeuroLex) and by electrophysiological property (using a newly developed ontology). We describe the techniques and challenges associated with the automated extraction of tabular electrophysiological data and methodological metadata from journal articles. We further discuss strategies for how to best combine, normalize and organize data across these heterogeneous sources. NeuroElectro is a valuable resource for experimental physiologists attempting to supplement their own data, for computational modelers looking to constrain their model parameters, and for theoreticians searching for undiscovered relationships among neurons and their properties.

## 1. Introduction

Brains achieve efficient function through implementing a division of labor, in which different types of neurons serve distinct functional and computational roles. One striking way in which neuron types differ is in their electrophysiology properties. Though the electrophysiology of many neuron types has been previously characterized and documented across decades of research, these data exist across thousands of journal articles, making cross-study neuron-to-neuron comparisons difficult.

Neurophysiology lacks a centralized resource where consensus data on basic physiological measurements from many neuron types and studies are accessible for reference and subsequent meta-analyses. For example, though it is common for neurophysiologists to measure and report neuronal measurements such as resting membrane potential and input resistance, there is not a public database which compiles this information. In other domains of neuroscience such efforts have made more progress. In the domain of neuroanatomical connectivity, information on connectivity between different brain regions is being compiled by experts at the Brain Architecture Management System project (BAMS) across thousands of publications (Bota et al., 2005). Parallel to this effort is the WhiteText Project, which addresses a complementary goal by algorithmically mining brain region connectivity statements from journal abstracts using biomedical natural language processing (bioNLP) methods (French et al., 2009, 2012). Similarly, in the domain of neuroimaging, the NeuroSynth Project has mined fMRI-based brain activation maps from published x,y,z coordinate data tables from thousands of neuroimaging publications (Yarkoni et al., 2011). These literature-based methods can be contrasted with projects such as NeuroMorpho.org (Parekh and Ascoli, 2013) and ModelDB (Migliore et al., 2003; Hines et al., 2004), which index neuron morphological reconstructions and computational models for simulating neuron activity by obtaining this information directly from investigators.

Success among these projects can be defined according to different criteria. Such criteria include completeness and comprehensiveness; for example, what percentage of relevant connectivity studies are indexed within BAMS? How many different neuron types are contained within the NeuroMorpho database? Alternatively, success can be defined in terms of the utility of these databases in driving subsequent research, like the use of BAMS as a resource for discovering relationships between brain region connectivity and gene expression (French and Pavlidis, 2011) or the use of NeuroMorpho to discover general scaling relationships among the morphologies of neuron types (Teeter and Stevens, 2011). Similarly, NeuroSynth is widely used by cognitive scientists as a starting point for designing functional imaging studies. Thus while these projects are not yet comprehensive and likely contain data records of varying quality, these resources may nevertheless be employed to draw novel inferences.

These projects are logically divided according to their methods for obtaining the source data: through the use of manual methods like expert curation or user contributions versus automated methods such as text-mining. Notably, these approaches differ in their scale and accuracy; while algorithmic methods can “scale-up” and be applied to arbitrary numbers of publications, they typically have a lower accuracy relative to human-curated content (French et al., 2009). This lower accuracy is often attributed to the rich lexical complexity of biomedical texts which often require considerable context and background knowledge to understand and parse (Dickman, 2003; Ambert and Cohen, 2012). The competing constraints of scale versus accuracy pose a challenge for large-scale compilation of neuroscientific data.

Here, we built a custom infrastructure framework for extracting electrophysiological measurements for specific neuron types from published neurophysiology articles. These measurements included properties such as input resistance and resting membrane potential, as well as associated metadata (i.e., article-specific methodological details). Our methods combine algorithmic literature text-mining, drawing from the approach used by NeuroSynth (Yarkoni et al., 2011) where neurophysiological measurements are primarily extracted from data tables, as well as manual curation, leveraging the background knowledge of domain experts. The resulting neurophysiology database, named NeuroElectro, can be interactively viewed and explored through a public web interface at http://neuroelectro.org.

## 2. Materials, methods, and results

### 2.1. Overview

**Figure 1:**
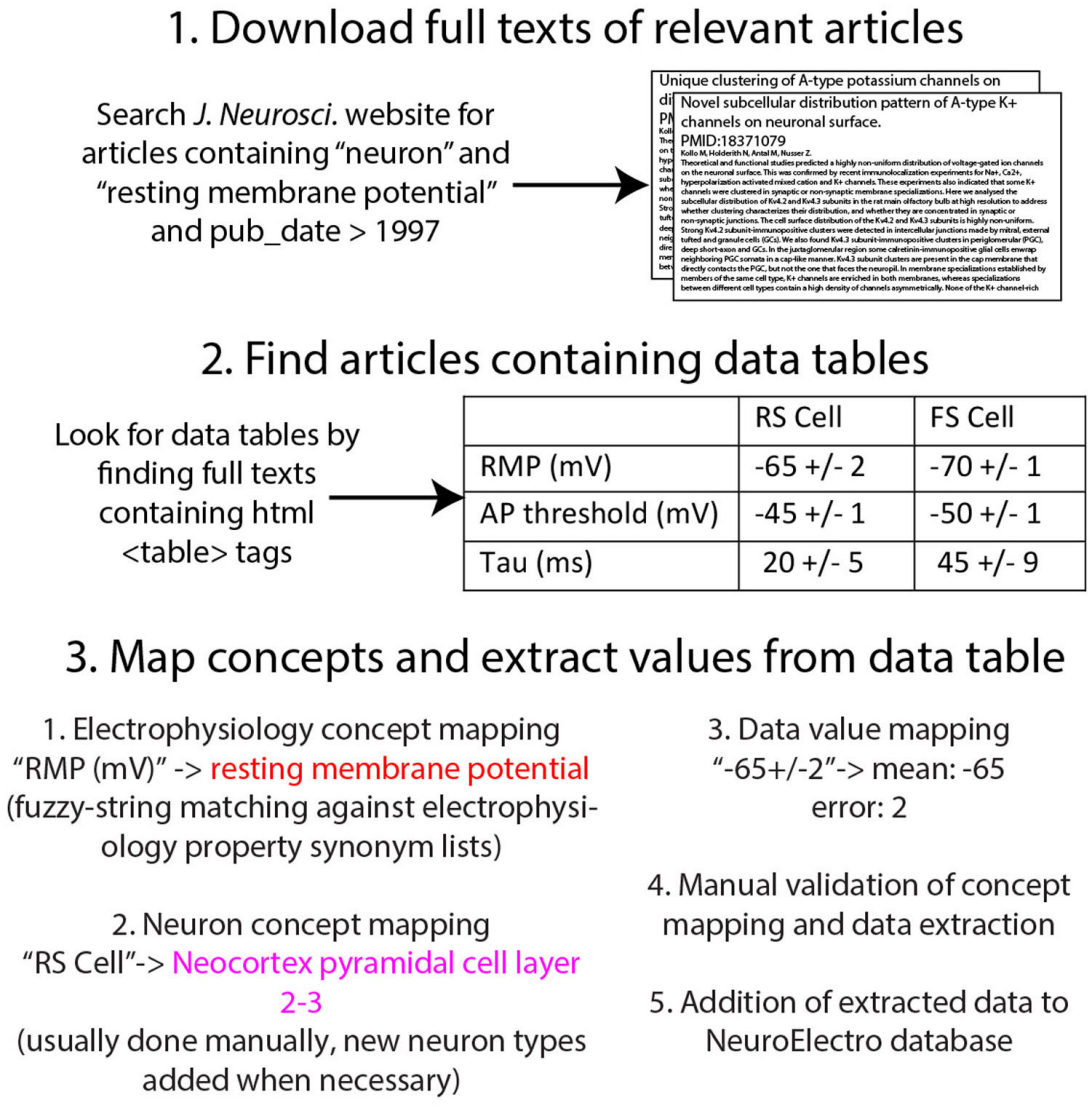
**Illustration of workflow for obtaining electrophysiological information from the research literature**.

We describe and validate our semi-automated methodology for obtaining neuronal biophysical measurements directly from published reports in the literature (summarized in Figure 1). After obtaining full article texts from publishers, we then used text-mining algorithms to identify concepts specific to electrophysiology and neuron types, which we then validated manually.

### 2.2. Article identification

We obtained electrophysiological data from 10 neuroscience specific journals (Table 1), which include: *Journal of Neuroscience, Journal of Neurophysiology*, and *Journal of Physiology* (among others). We selected these journals because they often devote a significant fraction of an article's main text, tables, and figures to detailed characterizations and summaries of intrinsic neuronal biophysical properties.

**Table 1:** **Statistics of journals represented in the NeuroElectro database**.

**Journal**	**Articles obtained**	**Validated**	**Not validated**
J. Neurosci.	19,002	104	560
J. Neurophysiol.	12,078	94	555
J. Physiol. (Lond.)	10,543	44	235
Neuroscience	3035	14	205
Eur. J. Neurosci.	2495	7	117
Brain Res.	3017	7	146
Neuron	1657	4	43
Epilepsia	463	2	23
Neurosci. Lett.	1468	2	34
Hippocampus	208	2	10

*Listing of journals and counts of articles downloaded (articles obtained), articles with published data tables containing neurophysiological information which has been manually validated by an expert curator (validated), and articles which likely contain information in a data table which has not yet been manually curated (not validated). Not validated articles are those which have at least four algorithmically assigned electrophysiological concepts within data tables*.

We obtained tens of thousands of potentially relevant full article texts directly from publisher websites. We first identified potential articles that were likely to contain information relevant to neuron biophysics using the native search functions provided within the journal websites and only downloaded articles containing in their full text any of a specific list of terms including “input resistance” and “resting membrane potential” (Figure 1). This pre-selection step allowed us to identify and download only articles that contained data relevant to our project. Upon identifying candidate articles, we then downloaded the full text of each potentially-relevant article as HTML; articles downloaded from the publisher Elsevier (e.g., *Neuron* and *Brain Research*) were downloaded as XML using the provided text-mining API and subsequently converted to HTML. We chose to work with HTML (as opposed to PDF or XML) because HTML provides a machine-readable markup of the article's content, allowing us easily to identify relevant elements within the article—such as data tables and the Methods section—using publicly available HTML-parsing tools (here we used the Beautiful Soup HTML-processing library implemented in Python: http://www.crummy.com/software/BeautifulSoup/bs4/doc/). Furthermore, because HTML is a single semi-structured standard used across publishers, we could write relatively generic HTML-processing algorithms applicable to content published across journals. Our focus on using HTML limits us to relatively newer articles—typically those published after 1996—because before this time most publications are only available as scanned PDF files. However, because the rate of publication across the field has grown exponentially, this HTML-available subset constitutes the majority of published neuroscience articles.

We stored the HTML-enhanced full text of each article in our database and associated each article with its corresponding PubMed ID (http://www.ncbi.nlm.nih.gov). These 8-digit IDs serve as publisher-independent unique identifiers for each article, and allow us to use PubMed-specific tools, such as a powerful API (i.e., PubMed eutils, http://www.ncbi.nlm.nih.gov/books/NBK25500/). For example, this API provides the ability to query each article's MeSH terms (MEdical Subject Headings) and returns basic methodological information such as animal species and strain.

### 2.3. Electrophysiological property identification

#### 2.3.1. Rationale for focusing on electrophysiological property extraction from data tables

**Figure 2:**
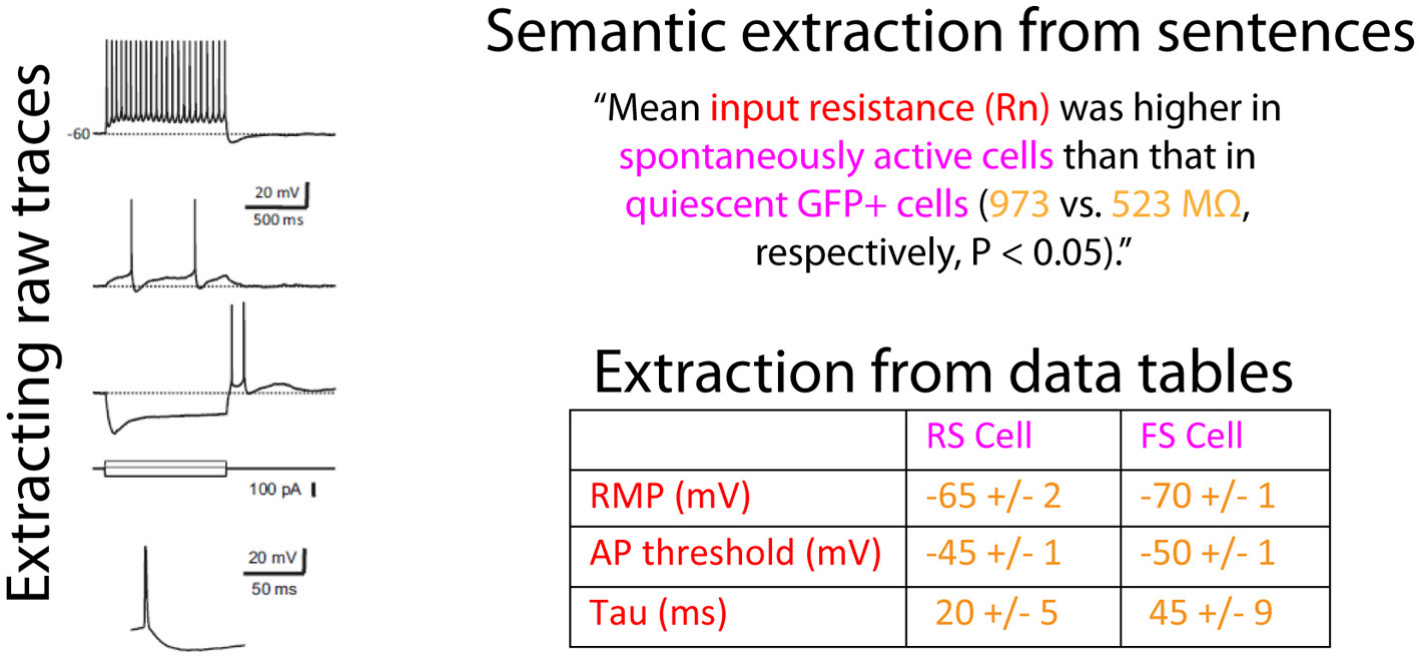
**Illustration of the sources within an article containing information relevant to neuron electrophysiological properties**. Data on neuronal electrophysiological properties are presented within article figures and raw traces, sentences within the article text, and formatted data tables. The raw traces and example sentence are from van Brederode et al. (2011) and are reproduced with permission from The American Physiological Society and the data table is a constructed example. Colored text indicates electrophysiological concepts (red), neuron concepts (pink), or neurophysiological data (yellow).

In order to algorithmically extract information on neuron electrophysiology from these articles, we needed to first specify the data types of interest. Our preference was to obtain as much detailed information about neuron electrophysiological properties as possible: ideally, this would include raw data corresponding to recorded electrophysiological traces. In mining information from articles, we were presented with multiple options (illustrated in Figure 2), including extraction from: (1) the text of the article including figure captions, (2) the figures of the article, or (3) data tables presented within the article. In addition to these, authors often submit supplemental materials and figures which also contain neurophysiological data.

Given the challenges in mining raw electrophysiological traces from figure images, we instead focused on obtaining information about basic neuronal electrophysiological properties, such as input resistances and resting membrane potentials. Though this information is often presented within the text of the article, it is usually presented in complex sentence structures that are difficult to accurately parse algorithmically. Published data tables, on the other hand, present a unique opportunity for electrophysiological data extraction, since common techniques exist for extracting information from structured tables (Yarkoni et al., 2011). Moreover, because tables succinctly summarize multiple attributes of a collected dataset, the effort of an expert curator can be put to best use when validating tables relative to validating content mined from article sentences or figure panels. While we estimate that only 5–10% of electrophysiology articles contain data tables, there is sufficient redundancy within the field (i.e., multiple investigators often publish articles on the same neuron type) that focusing on data tables nevertheless yields substantial coverage of electrophysiological properties across many major neuron types.

#### 2.3.2. Extracting information on electrophysiological properties

In extracting electrophysiological data, we took advantage of the fact that certain measurements are commonly made during intracellular recordings. For example, such recordings are commonly used to: (1) measure a neuron's resting membrane potential, (2) apply hyperpolarizing current injections for measurement of input resistance and membrane time constant, and (3) apply depolarizing current steps to evoke action potentials (spikes) and enable measurement of characteristics such as spike threshold, width, and amplitude.

We developed an electrophysiological lexicon comprising 28 measurements that we found to be commonly reported in the literature, largely based on previously published definitions (Toledo-Rodriguez et al., 2004; Ascoli et al., 2008). To account for subtle differences in terminology that authors use to refer to the same electrophysiological concept (e.g., resting membrane potential is often referred to as “rmp” and “V_*rest*_”), we also identified a common list of synonyms to map to each concept. Together, these electrophysiological concepts and their synonyms define a preliminary ontology for electrophysiological concepts (included in Supplemental Materials). Moreover, this physiological measurement ontology can serve as a scaffolding for a more in-depth ontology of electrophysiological investigations (e.g., Ontology for Experimental Neurophysiology, Bruha et al., 2013). The terms in our preliminary ontology are also indexed and defined within NeuroLex (http://neurolex.org, Larson and Martone, 2013).

To identify data corresponding to electrophysiological properties reported within a data table, we developed algorithms to search data table header elements and assess whether these elements corresponded to any of the electrophysiological concept synonyms in our ontology. We first identified table header elements by searching for table elements composed primarily of non-numeric characters. For each putative header element, we then used fuzzy string matching algorithms (implemented using the fuzzywuzzy library in Python: https://github.com/seatgeek/fuzzywuzzy), to assess the textual match between the header element and each of the electrophysiological synonyms. These fuzzy matching algorithms combine a number of string match metrics into a single “match value,” including whether a pair of strings completely match, contain matching substrings, or contain matching but misordered substrings. If the table header and electrophysiological synonym match value exceeded a specified threshold, the table header and corresponding row or column of numeric values were automatically mapped to the electrophysiological concept. Similarly, we mapped whole rows or columns to specific neuron types recorded during normotypic or “wild-type” conditions.

We then manually corrected cases where these algorithms misassigned an electrophysiological concept. For example, a common algorithmic mis-assignment was the case when an author used the string “EPSP amplitude” to refer to the electrophysiological concept excitatory post-synaptic potential amplitude. In these cases, our algorithms incorrectly mapped this string to “spike amplitude” because the former concept is not in our current ontology. In a test sample of 279 articles that were manually curated, we found that 78% of concept-matchings (901/1152) were identified correctly with no supervision, with the remainder manually corrected.

#### 2.3.3. Accounting for differences in electrophysiological definitions across investigators

By focusing on textually matching the electrophysiological terms in each table to a list of electrophysiological concepts, we are implicitly assuming that electrophysiological properties are measured in the same way by investigators across different articles. For example, the most common method that electrophysiologists use to measure a neuron's spike properties is to record from the neuron in current-clamp mode and apply peri-threshold depolarizing currents to evoke 1–2 spikes over several hundred milliseconds or more. The neuron's spike amplitude is then commonly measured by calculating the difference between the neuron's voltage at spike threshold and spike peak for the first evoked spike (e.g., Connors et al., 1982; Toledo-Rodriguez et al., 2004). However, experimental differences exist between how investigators measure and compute these properties; we divide these differences into roughly three categories: *protocol, calculation*, and *condition* differences. For example, investigators can use different experimental protocols to measure the spike amplitude, like evoking spikes using current steps much greater than rheobase current required to elicit a single spike (*protocol differences*). Additionally, the spike amplitude itself can be calculated in different ways, such as using the neuron's resting membrane potential as the baseline instead of the spike threshold (*calculation differences*). Furthermore, the value of spike amplitude that an investigator reports will also be affected by specific experimental conditions such as the animal species or age and recording solution temperature or contents (*condition differences*).

When manually curating the text-mined content for some of the most commonly reported electrophysiological properties, we accounted for an investigator's calculation of an electrophysiological measurement using an inconsistent methodology (e.g., protocol or calculation differences). We did so by normalizing such measurements to a common reference definition or removing such data when normalization was not possible. However, we note that we could not identify all of these cases (in particular: spike amplitude, input resistance, and membrane time constant), in part because investigators did not always explicitly define how these measurements were calculated within their article. We note that in cases where we pool measurements which are measured using inconsistent protocols or calculations, this will tend to add unexplained variance to our data set. Given these measurement inconsistencies, we provide our recommendations for how these electrophysiological properties should be reported in future investigations via our electrophysiology ontology (see Supplemental Materials).

### 2.4. Neuron type identification

#### 2.4.1. Using neuron types defined by neurolex

To extract physiological information specific to individual neuron types, we had to identify which neuron types were reported in each article. However, in many cases uniquely identifying the neuron type(s) reported in any given study and mapping these to a canonical “neuron type” is difficult. This difficulty arises in part because investigators use different criteria for classifying neurons, including electrophysiological, morphological, or molecular characteristics (Ascoli et al., 2008; Fishell and Heintz, 2013; Huang and Zeng, 2013).

To define canonical neuron types, we chose to use an existing list of approximately 250 neuron types and definitions provided by NeuroLex, a community-sourced, expert-defined collection of neuron types (http://neurolex.org; Shepherd, 2003; Hamilton et al., 2012; Larson and Martone, 2013). Moreover, we chose to use NeuroLex to keep our database consistent with existing resources and to enable future researchers to combine these resources seamlessly. NeuroLex also provides synonyms for each neuron type, which we utilized to identify the neuron type(s) in each article. In cases where a neuron type was investigated in the literature across multiple articles but not indexed within NeuroLex (e.g., cerebellar nucleus neurons), we manually added this neuron type to our database's listing and provided this neuron type to the NeuroLex neuron curators for incorporation (Gordon Shepherd, personal communication). Our specific criteria for identifying each of the neuron types reflected in the database are given in the Supplemental Materials.

#### 2.4.2. Identifying specific neuron types within an article

Because of the complexity in unambiguously identifying neuron types, we used a mixed text-mining and manual approach to map the neuron types studied in each article to canonical NeuroLex neuron types. First, we used text-mining algorithms to provide an initial “best guess” of the most likely neuron type. Specifically, we used a bag-of-words approach (Aldous, 1985) on the full article text. This approach ignores the serial structure of the words in the document and utilizes only the frequency of occurrence of each word within the document. We next compared the article's word-frequency histogram to the listing of neuron synonyms provided by NeuroLex, ranking all neuron types by their likelihood of being actually studied within that article. In comparison to articles that we manually curated, we found that this automated approach accurately identified the neurons studied in each article with an accuracy of 30% (120 of 399 total) and up to 55% when defining success as the studied neuron appearing as one of the top three neuron types suggested by the bag-of-words method. Because of the relatively low accuracy of an automated-only approach, we added a manual curation step where a curator identified the recorded neuron type using HTML drop down menus enriched by the bag-of-words search (e.g., Figure 4). As previously described, we mapped individual data table elements and corresponding rows or columns to specific neuron types recorded under normotypic conditions. We note that currently we only identify data from normotypic or “control” neurons represented in tables, but plan to identify data from additional conditions in future work (e.g., from pharmacologically manipulated or genetically modified animals).

### 2.5. Extraction of electrophysiological data values

**Figure 3:**
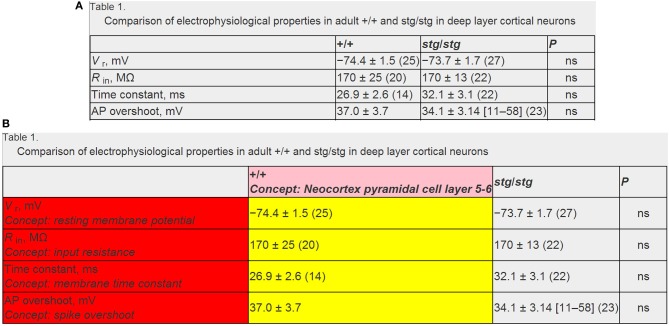
**Example data table illustrating mark-up and annotation of entities**. **(A)** Example published data table containing neurophysiological information. Data table from Pasquale et al. (1997) and is reproduced with permission from The American Physiological Society. **(B)** Same as **(A)**, but semantically marked up with algorithmic and manually curated annotations. Markups in red and pink indicate electrophysiological and neuron type concepts and yellow indicates extracted data measurements. Note that here the textual string “+/+” and “stg/stg” refers to the normotypic and manipulated condition, respectively. Panels **(A)** and **(B)** reflect screenshots taken from NeuroElectro web interface.

After identifying specific electrophysiological properties and neuron types reported in a data table (corresponding to row or column table headers), we then algorithmically extracted the data corresponding to the table intersection of these (Figure 3). We developed custom string regular expressions (Thompson, 1968) to parse the string corresponding to the numeric data. Specifically, we found that data strings were often of the form: “XX ± YY (ZZ),” where XX, YY, and ZZ refer to the mean, error term, and sample size (i.e., the “n”), respectively. Often, the number of replicates or error measurement were not reported or were reported in alternative ways within the table. Presently, the error term is not resolved as either a standard deviation or standard error measurement in the current version of NeuroElectro, but could easily be resolved in future iterations.

When designing our processing algorithms, we parsed data strings from right to left: first searching for data entities contained within parentheses, then for entities contained to the right of the ± term, and finally the remaining term which we assumed to refer to the mean term. We found that occasionally data were reported as “XX (LL–HH)”—where LL and HH indicate the lower and upper limits of a data range—and accounted for these cases similarly. We used regular expressions to identify entities such as digits, decimal signs, parentheses, and ± signs. We then converted the individual data elements which were encoded as textual strings of digits to double precision decimal entities before storing these into our database. Our focus here was primarily on parsing the mean value from a data record (i.e., summarizing the properties of a number of recorded neurons), but we also extracted and stored the error term and sample size where possible. Using these methods, we were able to extract 2176 electrophysiological values for 93 distinct neuron types within 279 articles.

### 2.6. Manual validation of automated data extraction

**Figure 4:**
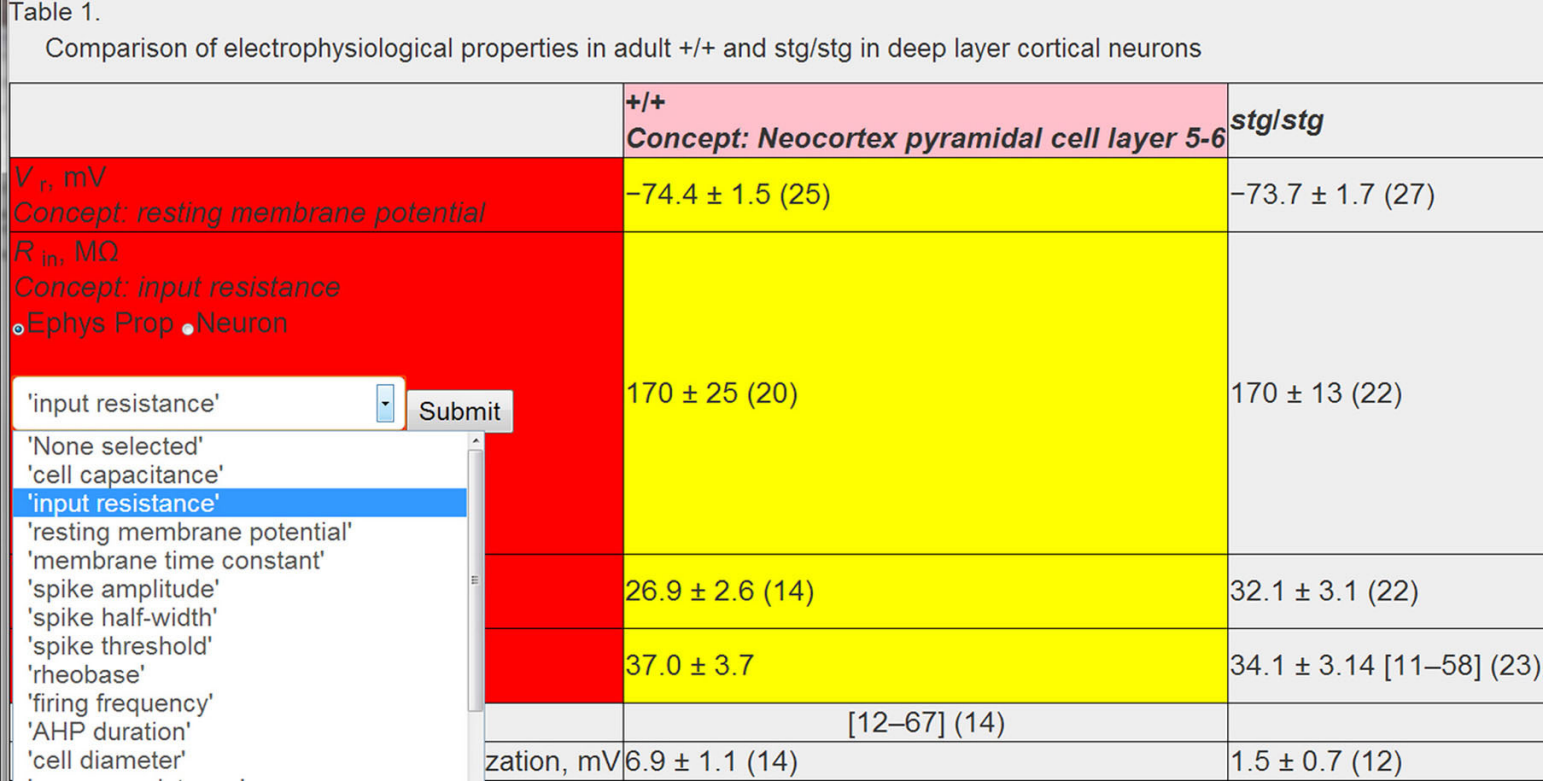
**Example of human validation of algorithmically assigned content**. All textual elements of a table are enhanced using HTML and javascript to allow for assignment of neuron or electrophysiological concepts using drop down menus. Example data table from Pasquale et al. (1997) and is reproduced with permission from The American Physiological Society.

Following these automated concept identification and data extraction steps, we manually validated associated concepts and corrected incorrect concept mappings as necessary. We developed custom-HTML and javascript code to allow human curators to graphically interact with downloaded HTML data tables and “mark-up” entities within the table (Figure 4). This code allows for textual based elements of the HTML table to be semantically annotated using drop down menus and text fields. Moreover, because annotation is implemented via user interfaces composed of interactive web pages and drop down menus, these user interfaces are simple enough to be utilized by other expert curators with little formal instruction.

### 2.7. Metadata identification

Given the strong relationships between experimental conditions, such as animal species or recording temperature, and electrophysiological measurements [e.g., input resistances are known to decrease when measured in neurons from older animals (Zhu, 2000; Okaty et al., 2009; Kinnischtzke et al., 2012)], we also identified information on article-specific experimental conditions by extracting this information primarily from each article's methods section. For each article, we found the methods section by developing custom HTML tag filters for each journal (e.g., common publisher-defined HTML tags for methods sections are “Methods” or “Experimental procedures”). For each metadata entity that we focused on (species, animal strain, electrode type, preparation type, liquid junction potential correction, animal age, recording temperature), we devised custom automated text searching methods to identify these based on combining regular expressions (Thompson, 1968) with PubMed MeSH terms (Table 2). In other words, rather than taking a machine-learning based approach and training classifiers (McCallum, 2002), we took a rule-based approach and developed custom rules for identifying metadata entities. For example, to identify whether the recording electrode's liquid junction potential was corrected for in the study (Neher, 1992), we searched for whether the character string “junction potential” was mentioned within the methods section and, if so, whether the sentence or phrase containing the term was explicitly negated (indicating that the junction potential was not corrected for). Here, we identified and parsed distinct sentences within the methods section using tools provided within the Natural Language Tool Kit in Python (Bird et al., 2009).

**Table 2:** **A partial listing of metadata attributes and extraction methodology**.

**Metadata concept**	**Values**	**Extraction method**	**Regular expression**	**MeSH term**
Species		MeSH term only		
	Rats			Rats
	Mice			Mice
	Guinea pigs			Guinea pigs
Electrode type		MeSH term + Regex		
	Patch-clamp		“Whole cell” or “patch clamp”	Patch-clamp techniques
	sharp		“Sharp electrode”	
Animal strain		MeSH term only		
	Fischer 344			Rats, Inbred F344
	Long-evans			Rats, Long-Evans
	Sprague-Dawley			Rats, Sprague-Dawley
	Wistar			Rats, Wistar
	C57BL			Mice, Inbred C57BL
	BALB C			Mice, Inbred BALB C
Preparation type		MeSH Term + Regex		
	*In vitro*		“Slice” or “*in vitro*”	
	*In vivo*		“*In vivo*”	
	Cell culture		“Culture”	Cell culture techniques
	Model		“Model”	Computer simulation
Junction potential		Regex		
	Not corrected		“Not junction potential”	
	Corrected		“Junction potential”	
				
Recording temperature		Regex		
	Continuous value		“Record… C” or “experiment C”	
	Room temperature		“Record room temperature”	
Animal age		Regex		
	Continuous value		Find digits near: “P#-#” or “P#-P#”	

*Metadata attributes are extracted through combining PubMed Medical Subject Heading terms (MeSH Terms) and custom regular expressions (Regex). Regular expression column (or MeSH Term column) indicates specific regular expressions (or MeSH terms) used for identifying metadata concept entities*.

**Figure 5:**
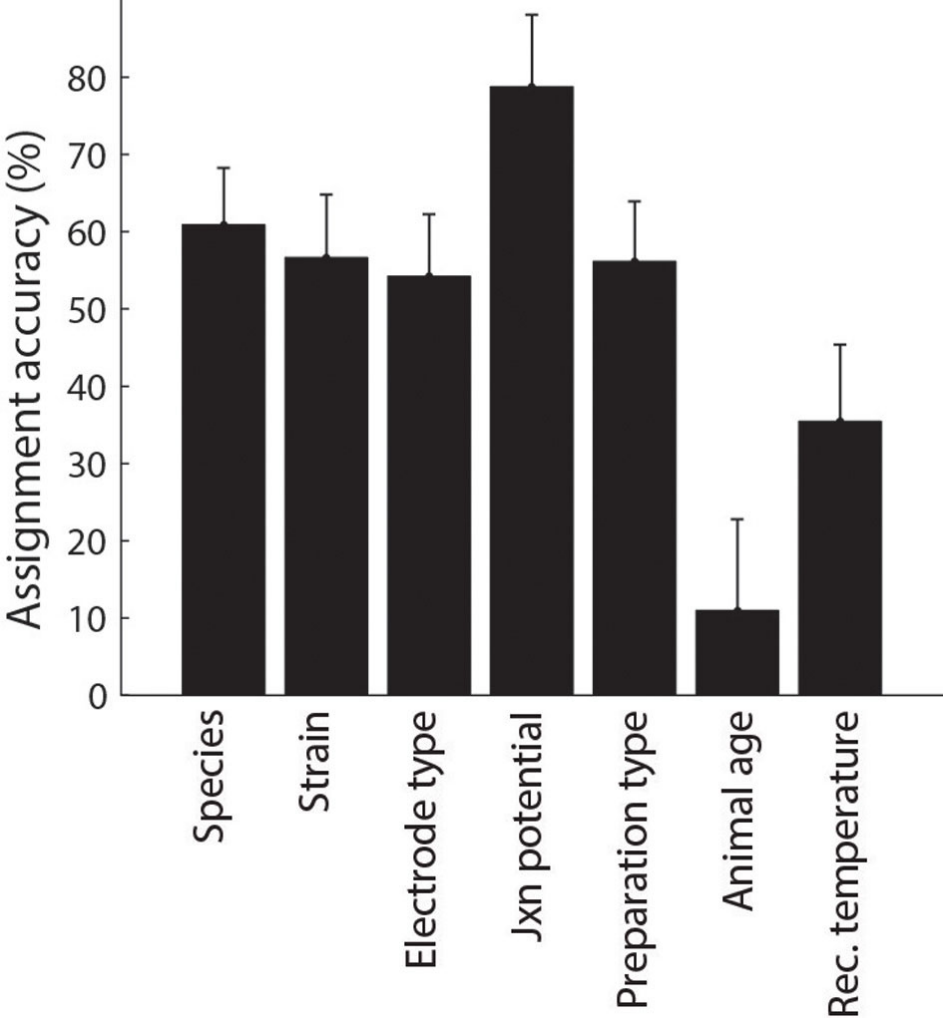
**Accuracy of metadata assignment using automated methods alone. Error indicate 95% binomial confidence intervals**.

Following automated identification of article metadata, we then manually checked each article to ascertain that algorithmically-tagged metadata was identified correctly and, as before, we corrected misidentified content as necessary through the use of custom HTML forms. We found that the mean accuracy of algorithmic metadata assignment was approximately 50% (Figure 5) and was typically lower for identifying continuous-valued metadata (e.g., animal age or recording temperature) relative to nominal metadata such as species and electrode type.

### 2.8. Object models and relational database

We stored extracted data and metadata using a relational database implemented in MySQL (http://dev.mysql.com/doc/refman/5.6/en/) built from a Python Django object model (https://www.djangoproject.com/). The object model contains classes for a number of fields, such as full article texts, electrophysiological properties, neuron types, synonyms, electrophysiological data values, and experimental metadata (Figure S1). A useful feature of the relational nature of the database is that it enables linking between classes (e.g., linking between neuron types and electrophysiological properties reported by a single investigator across multiple articles). This linking feature facilitates efficient and arbitrary querying of data; for example, querying for known electrophysiological data on olfactory bulb mitral cells recorded *in vitro* and published between the dates 2000 and 2004. For example, such a feature could be used to assess whether measurements of olfactory bulb mitral cells have changed as a function of time or are dependent upon whether the data are collected *in vitro* or *in vivo*.

### 2.9. Web application

The primary results of NeuroElectro are viewable at http://www.neuroelectro.org where the data can be interactively explored.

#### 2.9.1. Human interface

The web interface is organized around neuron types and electrophysiological properties. For example, each neuron type has its own webpage where extracted data corresponding to specific electrophysiological properties is graphically and interactively displayed (graphical plot interactivity implemented using the jqPlot javascript toolbox, http://www.jqplot.com/). Users can thus visualize the mean and variability of electrophysiological values across papers, view references plus experimental metadata, and easily navigate to primary data from specific papers. Furthermore, users can view electrophysiological data across all of the neuron types in the database—putting phenotypic properties of a given neuron type into the larger context of other neuron types located throughout the nervous system.

The web application also contains preliminary features to allow website visitors to contribute to the NeuroElectro resource. For example, users can suggest articles that contain electrophysiological data which are not already in the database. We also invite visitors to become “expert curators” for neurons of interest. In the future, we plan to build functionality that will allow investigators to upload raw and summary data, such as recorded voltage and current traces. In addition, we plan to continue mining the literature and adding neurophysiological measurements as they are published.

#### 2.9.2. API

An initial API (application programmer interface) providing public access to the electrophysiological data is described at http://neuroelectro.org/api/docs/. This RESTful API allows contents of the NeuroElectro database to be dynamically retrieved in JSON or XML format for utilization within external applications. For example, using the current API, a developer could build an application which dynamically queries NeuroElectro for all data corresponding to layer 2/3 neocortical pyramidal cells and then uses this data to constrain parameters for a Hodgkin–Huxley type neuron model (Hodgkin and Huxley, 1952). Example use cases of the current API (version 1) include:

- http://neuroelectro.org/api/1/n/ : Returns a list of all neurons with electrophysiological data indexed in NeuroElectro.
- http://neuroelectro.org/api/1/nedm/?nlex=sao830368389 : Returns a list of all indexed data on CA1 pyramidal cells (queried using the NeuroLex identifier for CA1 pyramidal cells, *sao830368389*).
- http://neuroelectro.org/api/1/nes/?e__name=Input+resistance: Returns a data record composed of the mean, standard deviation, and sample size n, summarizing input resistance measurements from cerebellar Purkinje cells based on all indexed articles in NeuroElectro database. Here the database query is performed using the textual strings for the electrophysiological and neuron type concepts.

Our future plans are to work with domain ontologists to further develop the existing API into a formal relational data format (RDF) specification, allowing further querying and extending of NeuroElectro into additional resources. All code used for the project is available at http://github.com/neuroelectro/neuroelectro.

## 3. Discussion

We have developed, applied, and validated a methodology and pipeline for extracting—from existing literature on cellular neurophysiology—measurements of basic biophysical properties from diverse neuron types throughout the nervous system. Currently, the NeuroElectro database contains 2344 manually curated electrophysiological measurements from 98 neuron types from 335 publications. Of these electrophysiological measurements, 2176 (93%) were obtained from 279 (83%) publications using the semi-automated approach described here. In addition, we machine-extracted and manually validated 1667 methodological conditions (metadata) from these publications. This represents the single largest collection of neurophysiological data ever compiled and represents a potentially valuable tool for scientific discovery.

### 3.1. Specific benefits provided by the semi-automated approach

One of the key advantages of the approach described here is that the automated pipeline identifies publications which are likely to contain content relevant to our domain area (i.e., measurements of neuronal biophysics). Thus a human needs only to manually curate the content first identified by the algorithms as being likely relevant, instead of having to identify the relevant content *de novo*. Moreover, the automated identification of neuron types in articles allows us to target manual curation efforts to publications likely to contain data from specific neuron types, such as neurons that are currently underrepresented in the database.

Given our laboratory's focus on olfactory circuits, we conducted a natural experiment to compare the efficacy of biophysical property extraction using these semi-automated methods versus traditional methods which do not make use of algorithmic text-mining as a pre-processing step. In a seven-hour curation session (evoking the classic American parable of John Henry versus the steam-powered hammer), a senior graduate student in our laboratory identified 91 electrophysiological measurements (focusing on resting membrane potential, input resistance, membrane time constant, spike amplitude, spike width, and spike threshold) from 35 articles for 7 olfactory bulb neuron types using only prior knowledge of which articles and investigators were likely to have reported such electrophysiological data. In a comparable seven-hour curation session using our semi-automated methods, a single curator (with similar expertise to the first curator) identified 551 electrophysiological measurements from 70 articles across 40 neuron types throughout the nervous system. Moreover, this comparison would likely tilt even more in favor of the semi-automated methods had the curators been less familiar with the primary literature.

### 3.2. Scalability of current approach

We note that multiple steps in our approach require manual intervention by an expert curator in order for electrophysiological measurements to be extracted with an acceptably low error rate. Namely, an expert curator needs to confirm which of the machine-identified candidate neuron types are recorded from in each article and where data from the normotypic or “control” states of these neurons are textually referenced within a data table. Moreover, given the current accuracy of the unsurpervised algorithmic assignment of electrophysiological concepts and experimental metadata (78% and 50%, respectively), these also need to be manually validated and corrected and normalized as required by an expert. Given the necessity of these manual steps, the scalability of our current approach is limited by our ability to manually curate this information or by our ability to improve the error rate of the automated methods. Despite this limitation, our current pipeline is still much faster than a purely manual one. The methodology could be further improved by correcting falsely matching entities (such as EPSP amplitude in section 2.3.2). These could be corrected by simply adding these valid concepts to the electrophysiolgical ontology. Moreover, these improvements would facilitate formally computing the sensitivity and specificity of these entity recognition methods.

### 3.3.Preliminary use of neuroelectro in scientific work

The NeuroElectro project is intended to facilitate scientific investigation by providing easy access to large quantities of data about neurons. Because the data is machine-readable, we have already begun to conduct several analyses that would not be possible without this resource. First, we have begun an investigation of the relationships between neurons as defined by the similarity of their electrophysiological properties. This information can be used to make predictions about as yet unmeasured properties. Second, we have begun to explore the relationship between patterns of gene expression [using both the Allen Brain Atlas (Lein et al., 2007) and single cell qPCR approaches] and electrophysiological properties of neurons. Third, we have begun automated testing of quantitative neuron models in concert with SciUnit (Omar et al., 2014), under the reasonable assumption that these models should be constrained by the available experimental data. These projects are described in manuscripts currently in preparation.

### 3.4. Extensions and improvements to the current semi-automated algorithms

Currently, neuron type identification is a critical bottleneck in our approach. One potential improvement would be to replace the non-specific bag-of-words approach we are currently using in favor of a bioNLP classifier-based approach (McCallum, 2002). Specifically, we propose adapting the named entity recognition methodology used by the WhiteText project for tagging brain regions mentioned in literature (French et al., 2009; French and Pavlidis, 2012) and first identifying spans of text likely to pertain to a neuron type before mapping these textual spans to a individual neuron type within the neuron ontology.

The approach described here is highly effective for extracting biophysical measurements presented within machine-readable data tables published within journal articles. However, the current requirement that these data tables exist in a machine parseable format, such as HTML or XML, limits this approach from being directly applied to older manuscripts, which are only available as scanned images. Existing approaches, such as optical character recognition technology (OCR; e.g., Ramakrishnan et al., 2012) may be applied toward this problem in the future.

Given the relatively low accuracy of the automated approach to identifying neuron types, there may be several avenues through which this process can be improved. For example, we note that the automated approach was particularly ineffective when the neuron type investigated within an article was not already described in NeuroLex or when the neuron had an insufficient list of synonyms associated with it. The current implementation of NeuroElectro also does not consider common neuron type acronyms (e.g., that olfactory bulb mitral cells are commonly referred to as “MCs”). Adding acronym and abbreviation identification to future iterations will thus likely improve the automated approach (Okazaki and Ananiadou, 2006; French and Pavlidis, 2012). Moreover, our current implementation of the bag-of-words algorithm would likely be enhanced via minor improvements, such as only identifying neurons using the text of the abstract or results and discarding text from the introduction or discussion. As neuron identification forms the major bottleneck in the scalability of NeuroElectro due to the requirement for manual curation, we plan to address this bottleneck in future revisions.

### 3.5. Future methods for data extraction

A more pressing issue with the current approach is its focus on extraction from data tables. We estimate that only 5–10% of published electrophysiological data is contained within tables, while the remaining 90–95% is presented within article text or figure images. Given our preference to obtain data in their most raw form, we initially considered extraction of data from figures, e.g., voltage traces of neuronal activity. However, digitizing article figures (presented by publishers as images) into a form that can be further analyzed presents multiple challenges. Though techniques and tools exist to digitize figures, substantial amounts of manual effort are required to employ them correctly, making this figure-based approach difficult to scale to increasing numbers of articles without also employing a large team of human curators. While automatically extracting measurements from figure images will likely prove challenging, our methods can likely be adapted to operate on article text, perhaps by making use of bioNLP methodologies currently used for relationship extraction in the identification of connected brain regions (French et al., 2012) or interacting pairs of proteins (Kim and Wilbur, 2011).

Future developments in machine extraction of data from the scientific literature will be of great benefit. These should include better semantic understanding of context, ranging from relatively unambiguous notations such as units, to syntax-parsing of free-form prose that relates objects of study to their reported properties. Much progress has been made by computer scientists in some of these areas, and more future engagement with their research should enable vastly more data to be extracted from the literature.

We believe that, if successful, the use of NeuroElectro will influence the practices of scientists writing papers and reporting results. Specifically, we recommend the usage of common standards and definitions for basic physiological measurements (Toledo-Rodriguez et al., 2004) and neuron types (Ascoli et al., 2008; Larson and Martone, 2013). Moreover, we advocate that, where possible, scientists report more basic physiological data overall and report such data using machine-parsable data tables. These recommendations could be made informally by journals (in particular, requested by reviewers during manuscript review) as well as by funding agencies. This change would make it easier for scientists to find and make use of data collected by others. Such a culture shift has the potential to make science function more effectively and efficiently to facilitate discovery.

## Funding

This work was supported by a National Science Foundation Graduate Research Fellowship and a R. K. Mellon Foundation Fellowship (to Shreejoy J. Tripathy), an Achievement Rewards for College Scientists Foundation Fellowship and NIDCD NRSA F31DC013490 (to Shawn D. Burton), NIDCD award F32DC010535 and NIMH award R01MH081905 (in support of Richard C. Gerkin), and NIDCD award R01DC005798 (to Nathaniel N. Urban).

### Conflict of interest statement

The authors declare that the research was conducted in the absence of any commercial or financial relationships that could be construed as a potential conflict of interest.

## Supplementary material

Figure S1 **Illustration of NeuroElectro relational database schema**.
