# Supplementary figures and images for: NeuroElectro: a window to the world's neuron electrophysiology data
Source: Front Neuroinform. 2014 Apr 29;8:40. doi: 10.3389/fninf.2014.00040 (PMC4010726; doi:10.3389/fninf.2014.00040)

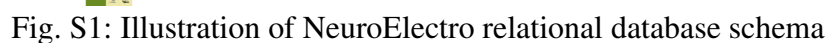

Supplement: Figure S1 — Illustration of NeuroElectro relational database schema. [file Presentation1.PDF]
